# Urinary Aspergillosis in a Patient with Systemic Lupus Erythematosus (SLE)

**DOI:** 10.1155/2023/5575300

**Published:** 2023-05-03

**Authors:** Mayara Gabriele Toledo, Hugo Rodrigues Alves, Isadora Cambruzzi, Laís Lopes Pires, Mariana Rossi, Ana Carolina Gonçalves Brito, Andrea D'Avila Freitas, Natalia Chilinque Zambão da Silva

**Affiliations:** ^1^Fluminense Federal University, Antônio Pedro University Hospital, Niterói, Rio de Janeiro, Brazil; ^2^Niterói Hospital Complex, Rio de Janeiro, Brazil; ^3^Niterói Hospital Complex, Niterói, Rio de Janeiro, Brazil; ^4^Pathologist at Diagnostics of America (DASA), Niterói Hospital Complex, Brazil; ^5^Infectologist at Niterói Hospital Complex, National Institute of Infectology Oswaldo Cruz Foundation, Rio de Janeiro, Brazil; ^6^Department of Clinical Medicine, Fluminense Federal University, Antônio Pedro University Hospital, Niterói, Rio de Janeiro, Brazil

## Abstract

Aspergillosis is an opportunistic mycosis that generally affects the lungs. The fungus was cleared by the immune system of a healthy host. Extrapulmonary forms are very rare, and there are few reports of urinary aspergillosis. In this case report, we describe a 62-year-old woman with systemic lupus erythematosus (SLE) with complaints of fever and dysuria. The patient had recurrent episodes of urinary tract infection and several hospitalizations. A computed tomography revealed an amorphous mass in the left kidney and bladder. After partial resection of the material was referred for analysis, Aspergillus infection was suspected and confirmed by culture. Successful treatment with voriconazole was provided. Diagnosis of localized primary renal Aspergillus infection in a patient with SLE requires careful investigation due to its benign presentation and lack of associated systemic clinical features.

## 1. Introduction

*Aspergillus* spp. are ubiquitous saprophytic fungi that comprise more than 200 species, most of which are nonpathogenic. *Aspergillus (A) fumigatus* is the most frequent cause of human Aspergillosis, in addition to *Aspergillus flavus* and *Aspergillus nigri* [1]. Numerous individual factors may predispose someone to Aspergillus colonization and pathogenicity but becoming immunocompromised appears to be a major risk factor [2–4]. Clinical presentations of aspergillosis are divided into noninvasive and invasive diseases. Invasive aspergillosis may present as pulmonary or extrapulmonary dissemination. Aspergillus usually affects the respiratory tract and, very rarely, other sites [3]. Aspergillosis limited to the urinary tract is a rare disease. Three different patterns of renal Aspergillus infection have been described, namely, disseminated Aspergillosis with haematogenous renal involvement, Aspergillosis of the renal pelvis with bezoars formation, and ascending panurothelial Aspergillosis [5]. We hereby report an unusual form of urinary tract aspergilloma in an immunocompromised patient with systemic lupus erythematosus (SLE). She was successfully treated with voriconazole, associated with surgical intervention.

## 2. Case Presentation

A 62-year-old female, with a past medical history of obesity, diabetes mellitus, systemic arterial hypertension, hypothyroidism, and SLE complicated with transverse myelitis and paraplegia, was admitted to the emergency department in June 2016 with dysuria, low back, and abdominal pain and fever. Urinalysis and laboratory tests were performed, and she was treated for a urinary tract infection (UTI) with culture-guided antibiotic therapy. After this presentation, for three years, the patient returned to the unit more than 10 times with the same complaints, being repeatedly diagnosed with recurrent urinary tract infection. During several hospitalizations for venous treatment and investigation of the condition, the patient required indwelling bladder catheters (IBC). Throughout the returns to the unit for the repeat condition, numerous antibiotics were used, guided by culture: Ertapenem (September 2018) and Nitrofurantoin (May 2019).

In August 2019, she presented Ureterolithiasis and hydronephrosis complicated with pyelonephritis. On this occasion, the patient was treated with amikacin. Then, in March 2020, she underwent rigid ureterolithotripsy with removal of the ureteral calculus and placement of a double-J catheter. The device was spontaneously expelled. In April of the same year, during a routine imaging, a mass was seen in renal topography. The patient underwent mass extraction and all negative laboratory investigations were negative.

One month after removing the renal mass, the patient returns to the hospital with new urinary complaints. Computed tomography (CT) showed a collection in the renal store and subcutaneous tissue in this region that was drained. *Proteus spp*. ESBL was isolated in urine culture and started ertapenem therapy. From May to August 2020, she remained asymptomatic, although in August 2020, the patient was admitted again with urinary complaints. The CT scan performed showed recurrence of the calculus on the left, with obstruction and dilatation upstream. A new procedure for ureterolithotripsy with the removal of the ureteral calculus and placement of a double-J catheter was performed.

**Figure 1:**
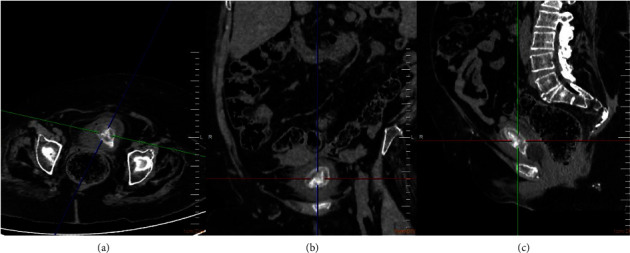
Pelvic CT images (axial (a), coronal (b), and sagittal sections (c)) showing an amorphous dense oval mass in the bladder.

In December 2020, the patient was presented with IBC obstruction, abdominal pain, and cloudy urine. Nephrostomy, armed cystoscopy, and double J catheter exchange were performed. Still presenting with repeated UTIs, in August 2021, the patient was admitted again with pyuria, urine culture with *Pseudomonas aeruginosa*, sensitive to meropenem. In September of the current year, a CT was performed, revealing an amorphous image with calcium density and local obstruction in the left kidney, with fluid infiltration in the left perineal space, besides the identification of a dense oval mass in the bladder, as seen in Figure 1. In the same month, the patient underwent armed cystoscopy, percutaneous left nephrostomy, complete resection of the bladder mass, and drainage of purulent renal secretion. Analysis of the secretion showed it to be polymicrobial.

**Figure 2:**
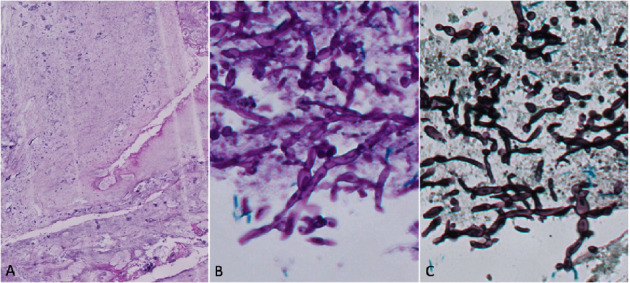
Vesical mass: (a) haematoxylin and eosin stain (H&E), 10x. Vesical mass sections showing clear eosinophilic amorphous material and some structures suggestive of hyphae (b) periodic acid-schiff (PAS) stain, 40x. Fungal septate hyphae with dichotomous branching, suggesting *Aspergillus* species. (c) grocott's methenamine silver stain, 40x.

Due to the history of repeated use of antimicrobials without resolution of the condition, associated with the presence of a mass, after analysis of material, *Aspergillus* infection was suspected and a specific culture was requested, like histopathological analysis identified *Aspergillus*. According to Figure 2.

Intravenous voriconazole was initiated, with improvement, after exchange of voriconazole for oral route was performed for continuity of treatment at home. Treatment was performed for 21 days. After this period, a new urine culture was performed for negative fungi. Control urinary computed tomography and ultrasound without evidence of fungal ball. The patient is being followed up with no recurrence of the condition. Laboratory data at the start of treatment, midtreatment, and end of treatment are described in Table 1.

## 3. Discussion

Renal aspergillosis is a rare infectious condition with few reports in the literature and the prevalence of it is unknown. It usually occurs through haematogenous dissemination, although it may result from contamination after kidney surgical procedures. The main clinical manifestations of renal aspergillosis reported are low back pain, low fever, elimination of whitish lumps in the urine, and no oliguric acute obstructive renal failure [6]. The presence of *Aspergillus* spp. urine culture does not imply infection, since contamination of the material is possible. This could be explained by *Aspergillus'* wide distribution in the environment, present in water, soil, air, and any location with organic debris [6, 7].

Early diagnosis of aspergillosis is difficult and requires a high level of suspicion. Unfortunately, there are several reasons for the delay or nondiagnosis of extrapulmonary disease, especially in SLE patients, such as the mimicry of other diseases promoted by SLE, concomitant presence of bacterial infection, lack of experience, and insufficient surveillance [7–9].

About 50% of SLE patients will develop some serious infectious disease over the course of the disease. Moreover, infections are responsible for 25% of SLE deaths. Invasive aspergillosis in patients with Lupus usually occurs in cases with high disease activity according to the SLE Disease Activity Index score, high-dose corticosteroid use, granulocytopenia, and other immunosuppressive treatments [6].

Management of renal aspergillosis is based on urologic and drug approach as treatment [6]. The timing of medications is unknown and is managed according to patient response [7, 9]. Although the use of voriconazole, posaconazole, itraconazole, and echinocandins has been described by literature as low urinary concentration, the patient was treated satisfactorily with voriconazole. Nephrectomies should be performed only as the last option [10].

Although it presents lower urinary concentration, it is ideal that during the use of voriconazole, its serum concentration should be dosed. It is a drug metabolized by cytochrome P450, and the maintenance of adequate therapeutic serum levels is essential to avoid adverse effects such as hepatic and neurological toxicity [11, 12]. The serum level presented by the patient was 3.3 mg/L, within the therapeutic range. Serum dosage is formally indicated for (i) patients with serious fungal infections, (ii) critically ill patients admitted to intensive care units, (iii) patients with liver cirrhosis or hyperbilirubinemia because of the reduced clearance of voriconazole, (iv) transplant recipients receiving voriconazole for prophylaxis against fungal infections, and (v) patients undergoing long-term outpatient treatment or prophylaxis [12]. It is recommended that the dosage be performed in a sample collected from the third day of medication use. The collection should be performed immediately before the next antifungal dose (within 30 minutes) [13].

## 4. Conclusion

In summary, early diagnosis and treatment of aspergillosis are challenging in clinical practice. In the presence of a prolonged urinary tract infection unresponsive to antibiotic therapy and a patient with risk factors, especially immunosuppression, invasive fungal disease should be considered. The complete clinical reasoning aids the earlier detection of the disease, and with this, advances the treatment.

The case highlights the importance of suspecting fungal disease in a patient with SLE with recurrent urinary tract infections and a history of urinary tract instrumentation. Fungal infections should be suspected in patients with lack of response or worsening despite antibiotics. The biopsy of the bladder mass was fundamental for the diagnosis because routine urinalysis may produce negative results due to intermittent excretion of the fungus in the urine.

## Figures and Tables

**Table 1:** Laboratory markers at diagnosis (september 2021), midtreatment (november 2021), and end of treatment (december 2021).

	September 2021	November 2021	December 2021	Reference range
The red blood cell indices				
Erythrocytes	2.81·10^6^/*μ*L	3.22·10^6^/*μ*L	3.75·10^6^/*μ*	4.00–5.20·10^6^/*μ*L
Hemoglobin	7.2 g/dL	8.3 g/dL	9.4 g/dL	12.0–16.0 g/dL
Haematocrit	22%	25%	29%	36.0–46.0%
Leukogram	10.500/*μ*L	9.500/*μ*L	10.100/*µ*L	4.500–11.000/*μ*L
C-reactive protein	15.07 mg/dL	13.56 mg/dL	9.29 mg/dL	<0.30 mg/dL
Dosage voriconazole		3.3 mg/L		2.0–5.0 mg/L

## Data Availability

The health record data used to support the findings of this case report are restricted in order to protect patient privacy. Appropriate, certain health record data are included verbatim within the article. This case report also provides a discussion of urinary aspergillosis. The data used in the discussion were found in peer-reviewed journals and previously published care reports. Appropriate citations and references are included within the article.
